# A Framework for Identifying Selective Chemical Applications for IPM in Dryland Agriculture

**DOI:** 10.3390/insects6040988

**Published:** 2015-12-16

**Authors:** Paul A. Umina, Sommer Jenkins, Stuart McColl, Aston Arthur, Ary A. Hoffmann

**Affiliations:** 1 School of BioSciences, The University of Melbourne, Parkville, Victoria 3010, Australia; E-Mails: sommerjenkins@hotmail.com (S.J.); ary@unimelb.edu.au (A.A.H.); 2 **cesar** Pty. Ltd, 293 Royal Parade, Parkville, Victoria 3052, Australia; E-Mails: samccoll@gmail.com (S.M.); astonarthur@bigpond.com (A.A.)

**Keywords:** pesticide, pests, toxicity, invertebrate, agro-ecosystems

## Abstract

Shifts to Integrated Pest Management (IPM) in agriculture are assisted by the identification of chemical applications that provide effective control of pests relative to broad-spectrum pesticides but have fewer negative effects on natural enemy (beneficial) groups that assist in pest control. Here, we outline a framework for identifying such applications and apply this framework to field trials involving the crop establishment phase of Australian dryland cropping systems. Several chemicals, which are not presently available to farmers in Australia, were identified as providing moderate levels of pest control and seedling protection, with the potential to be less harmful to beneficial groups including predatory mites, predatory beetles and ants. This framework highlights the challenges involved in chemically controlling pests while maintaining non-target populations when pest species are present at damaging levels.

## 1. Introduction

Worldwide, there is recognition that chemical usage patterns in agriculture are unsustainable and that methods to reduce the use of broad-spectrum pesticides need to be developed so that the role of natural enemies of pests can be expanded [1,2,3]. Populations of natural enemy (beneficial) groups are invariably higher on farms where pesticide use is minimized or pesticides are removed completely [4,5]. There has been much progress in identifying the impact of pesticides on non-target beneficial organisms, such as through the International Organization for Biological Control (IOBC), who develop standard methods (ranging from laboratory through to field experiments) for testing the side effects of pesticides on natural enemies [6]. Despite this knowledge, there is still reluctance by the majority of farmers to use alternatives to broad-spectrum pesticides [7,8]. This situation is certainly true in Australia, particularly in the grains industry, which is one of the largest primary industries, with exports worth almost AU$6 billion annually and over 35 million ha planted each year [9]. Invertebrate pests are a significant cost to Australian grain production, not only in terms of direct crop damage and control but also indirect costs as vectors of numerous plant diseases [10,11]. Pesticides are currently the main method of control against invertebrate pests attacking grain crops, mainly due to their low cost, effectiveness and ease of application [12,13]. These chemicals are often applied prophylactically prior to and/or soon after sowing to protect small gross margins and as a safeguard against pest infestations [13,14,15].

Pests that attack Australian dryland crops at seedling establishment are particularly damaging and responsible for millions of dollars in lost production and chemical control costs each year [13]. *Halotydeus destructor* Tucker (redlegged earth mite) and the *Penthaleus* species complex (blue oat mites) are considered to be among the most important establishment pests of Australian grain crops [14,16,17]. Redlegged earth mites and blue oat mites are active during the winter-cropping season in Australia, with an inactive diapause period over the summer months [17,18]. They are widely distributed across southern Australia and extremely polyphagous, attacking a broad range of plant types, including cereals, oilseeds, pulses, and a variety of pasture species [16,17,19]. As in the case of other plant-feeding mites [20], the current heavy reliance on broad-spectrum pesticides is not a sustainable practice and can lead to resistance problems [21], emergence of secondary pests [22] and loss of natural enemies [23,24]. High resistance levels to synthetic pyrethroids have already been observed in *H. destructor* after continuous exposure to these chemicals in the field [21,25]. Additionally, there is a high likelihood that a number of broad-spectrum chemicals will be removed from the market as a result of international pesticide legislation in reaction to potential hazards to health and the environment. Endosulfan has already been banned in Australia as a result of this [26], and several other chemical groups are currently under review [27]. There is a need to expand chemistry available to growers, ideally focusing on selective (also referred to as “soft”) pesticides and seed dressings that have reduced impacts on beneficial invertebrates and fit within a broader Integrated Pest Management (IPM) program.

There are many selective pesticides currently registered for use within the horticulture, viticulture and Australian cotton industries. However, there have been few attempts to move from using conventional broad-spectrum pesticides to more selective pesticides within the broadacre grains industry, even though selective chemicals can help maintain populations of natural enemies and other beneficial invertebrates that provide important ecosystem services in terms of pest control, seed dispersal, pollination of plants and enhancing soil health [28,29]. Selective chemicals are often more expensive in the first instance but can become economically viable when considering their long-term benefits [30]. Unfortunately, the benefits of selective chemicals have typically only been assessed through laboratory bioassays on a few species; the relevance of these assays to field conditions is rarely assessed [31].

**Figure 1:**
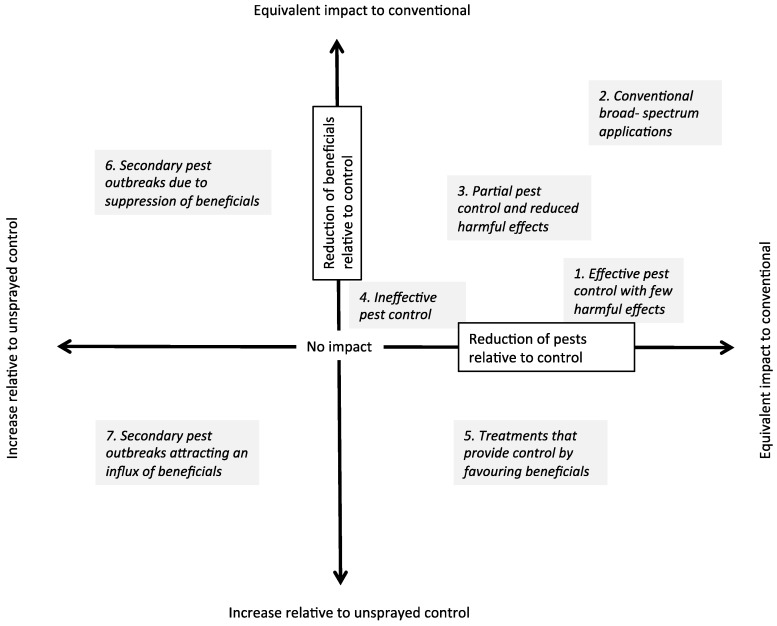
A framework for assessing the impact of new chemical applications on pest and beneficial invertebrates relative to a conventional chemical treatment and unsprayed treatment. The x-axis represents the effectiveness of the application for controlling a target pest relative to a control treatment. At the intersection with the y-axis there is no control of the pest (*i.e.*, pest numbers are equivalent to an unsprayed control). The y-axis represents the relative reduction of the application on beneficials relative to the unsprayed control. Ideally, new applications will be less harmful but still provide control (1) at similar levels to the conventional treatment, (2) whereas many new chemicals are expected to provide reduced levels of control but also reduced levels of harm (3). Pest numbers can also increase (to the left of the origin) if there are idiosyncratic effects (6,7).

One approach for testing the impact of new chemical applications might involve a comparison of effects on pests and beneficial groups to both a conventional (broad-spectrum) treatment and a treatment where no sprays are applied (Figure 1). This field chemical evaluation (FCE) framework would allow for the effectiveness of new chemical applications against pests to be contrasted to current treatments and for the relative benefits of new applications to be expressed relative to a situation where no chemicals are applied. Ideally, new chemical applications would provide effective control but reduced impact on beneficial groups (1 in Figure 1). However, control might be less effective even if there are fewer harmful effects (3 in Figure 1). A number of idiosyncratic outcomes are also possible due to interactions between chemicals, pests and non-target beneficials. For instance, pest numbers might increase if chemicals are particularly harmful to an important group of beneficial organisms (6 in Figure 1), while secondary pest outbreaks could attract an influx of beneficials into the system (7 in Figure 1).

The efficacy of several selective and broad-spectrum pesticides against mite pests of Australian grain crops has been tested in laboratory bioassays [32], which showed that a number of chemicals have potential to provide control against *H. destructor* and *Penthaleus* spp., although no single pesticide was found to be effective against all species tested. Furthermore, Jenkins *et al.* [33] tested the impact of broad-spectrum pesticides and several selective pesticides on these pests and non-target invertebrates under field conditions in wheat and canola, and found that selective chemicals generally had fewer negative effects on non-target species than the broad-spectrum chemicals, although these patterns were inconsistent among invertebrate groups. In this study, we extend the findings of Jenkins *et al.* [33] by examining the effectiveness of several selective pesticide treatments in the field, as well as exploring some non-chemical approaches that could work hand-in-hand with selective pesticides (e.g., weed cover treatment). These were compared directly with broad-spectrum chemicals commonly used by Australian farmers, allowing treatments to be considered within the FCE framework.

## 2. Experimental Section

### 2.1. Trial Sites

During 2009 and 2010, four field trials were established to examine the efficacy of selective pesticides against crop establishment pests and non-target invertebrates in Australia. Two trials were conducted in 2009; one site was located near Inverleigh, Victoria (38°09'08'' S, 144°00'36'' E) and the other at Rossbridge, Victoria (37°28'54'' S, 142°50'25'' E). In 2010, the trials were repeated at the Inverleigh and Rossbridge sites. Locations for trials were chosen specifically to target field sites with low history of pesticide use and moderate numbers of crop invertebrates.

All trials were designed in a randomized block arrangement with four blocks consisting of six plots per block. Each plot measured 20 m × 20 m, with a 5 m buffer of bare ground separating blocks to limit the movement of invertebrates between plots. In 2009, plots were sown with canola (*cv.* CB™ Argyle) at a rate of 4 kg/ha at Inverleigh and 5 kg/ha at Rossbridge. In 2010, plots were sown with wheat (*cv*. Bolac) at a rate of 70 kg/ha at Inverleigh and 88.8 kg/ha at Rossbridge. In all trials, treatments were allocated four replicate plots. Sowing rates were chosen based on local farming practice.

### 2.2. Chemical Treatments

Table 1 outlines the treatments applied at each trial site. Broad-spectrum pesticides were applied at the recommended field rate for *H. destructor*, which was the main pest present at all sites. The selective pesticides tested in this study are not currently registered for use in broadacre grain crops within Australia, therefore treatments were applied at rates used in other industries and/or recommended directly by agrichemical manufacturers. A low rate of dimethoate was included as a treatment in one trial; although dimethoate is an organophosphorus (and “broad-spectrum”) chemical, it has previously been found to have little negative impact on some non-target invertebrates [34]. Thus for the purposes of this study, we initially categorized dimethoate (when applied at a low field rate) as a selective chemical. All chemical treatments were reported to have toxicity against mites, the target group of pests in this study.

**Table 1:** Chemical treatments applied at each trial site.

Trial Site	Treatment Name	Chemical Trade Name	Active Ingredient(s)	Rate (mL/ha)	Treatment Date(s)	Gaucho^®^ Seed Coating
Inverleigh 2009	Broad-spectrum *	Talstar and Le-mat	bifenthrin (250 g/L) and omethoate (290 g/L)	40 and 100	02/06/2009 and 24/06/2009	No
	Thiamethoxam/abamectin	Columbus	thiamethoxam (72 g/L)/abamectin (36 g/L)	300	24/06/2009	Yes
	Spinosad	GF-1587	spinosad (100 g/L)	83.6	24/06/2009	Yes
	Weed cover	-	-	-	-	Yes
	Imidacloprid	-	-	-	-	Yes
	Untreated control	-	-	-	-	No
Rossbridge 2009	Broad-spectrum *	Talstar and Le-mat	bifenthrin (250 g/L) and omethoate (290 g/L)	40 and 100	04/06/2009 and 16/07/2009	No
	Diafenthiuron	Pegasus	diafenthiuron (500 g/L)	400	16/07/2009	Yes
	Paraffinic oil	Canopy	paraffinic oil (792 g/L)	5000	16/07/2009	Yes
	Weed cover	-	-	-	-	Yes
	Imidacloprid	-	-	-	-	Yes
	Untreated control	-	-	-	-	No
Inverleigh 2010	Broad-spectrum	Talstar	bifenthrin (250 g/L)	40	22/06/2010	Yes
	Thiamethoxam/abamectin	Columbus	thiamethoxam (72 g/L)/abamectin (36 g/L)	400	22/06/2010	Yes
	Dimethoate	Danadim	dimethoate (400 g/L)	55	22/06/2010	Yes
	Weed cover	-	-	-	-	Yes
	Imidacloprid	-	-	-	-	Yes
	Untreated control	-	-	-	-	No
Rossbridge 2010	Broad-spectrum	Talstar	bifenthrin (250 g/L)	40	29/06/2010	Yes
	Diafenthiuron	Pegasus	diafenthiuron (500 g/L)	400	29/06/2010	Yes
	Thiamethoxam/abamectin	Columbus	thiamethoxam (72 g/L)/abamectin (36 g/L)	400	29/06/2010	Yes
	Weed cover	-	-	-	-	Yes
	Imidacloprid	-	-	-	-	Yes
	Untreated control	-	-	-	-	No

* These treatments had two broad-spectrum applications; a bare earth foliar application of bifenthrin was applied post sowing/pre-emergence and a second application of omethoate was applied at the same time as the selective treatments.

Pesticides were applied once canola seedlings had reached the first true leaf stage and wheat seedlings had reached the one leaf stage (approximately Zadok’s 11). These are known susceptible crop stages when pesticides are often applied to control invertebrate pests within Australia [13,17]. For the broad-spectrum treatments at the 2009 sites, a “bare-earth” application was applied on the day of sowing (well before the emergence of crop seedlings), followed by a second application once the crop had emerged (post sowing) (see Table 1); this is common practice when sowing canola in Australia [14]. The second spray was applied at the same time as the selective pesticides. The untreated control plots were left unsprayed. In addition to investigating different chemical treatments, we also explored the role that alternative host plants play in minimizing pest-feeding damage to emerging crop seedlings. To do this, we included a weed cover treatment in which plots did not receive a pre-sowing herbicide application that was applied to all other plots as per local practice. The weeds within these plots consisted mostly of capeweed (*Arctotheca calendula*), clover (*Trifolium* spp.) and ryegrass (*Lolium* spp.).

All chemical treatments were applied in a total volume of 100 L/ha using a trailing boom spray (UniBoom model 600 L TR) with TeeJet (Glendale Heights, IL, USA) flat fan nozzles (02-fine) at 3 bar pressure. Chemical sprays were applied in dry conditions when average wind speed was below 15 km/h. At Rossbridge and Inverleigh, treatments were assigned the same plots over the two-year period (*i.e.*, the untreated control plots in 2009 were also the untreated control plots in 2010). In 2009, the canola seed sown in several field plots was coated with imidacloprid (Gaucho^®^ 600, Bayer CropScience, Melbourne, Australia) at the recommended rate of 400 mL/100 kg (Table 1). However, the seed used in the untreated control plots and the broad-spectrum plots were left untreated. In 2010, all wheat seed, except for the untreated control plots, was coated with imidacloprid at a rate of 240 mL/100 kg (Table 1). Pesticide seed coatings were incorporated because they offer protection to crop seedlings from moderate pest densities and can allow foliar pesticide applications to be delayed. This complements pesticides with systemic and translaminar properties, which are common across many of the selective chemicals examined.

### 2.3. Invertebrate Sampling

A combination of vacuum sampling and pitfall traps was used to assess the abundance of invertebrates across plots, both prior to, and after, chemical applications. These techniques are commonly used to assess the densities of ground-dwelling invertebrates in the field [35,36,37]. Once samples were collected and brought back to the laboratory, they were first sorted to order level using a stereomicroscope with 20× to 100× magnification. Key pest and non-target invertebrates were then further identified by sorting them into family and species levels.

Vacuum sampling was performed via suction using a Stihl SH55 blower vacuum (Andreas Stihl AG & Co. KG, Waiblingen, Germany), with four samples randomly taken per field plot. For each sample, we vacuumed the soil surface and vegetation within a 0.09 m^2^ frame over a period of 10 s. Suction samples were taken using a 100-micron fine cup sieve fitted on to the end of the vacuum spout, with the contents transferred to vials containing 70% ethanol. Pitfall traps consisted of a plastic vial 11 cm deep and 4.5 cm in diameter placed into a polyvinyl chloride (PVC) sleeve in the ground so the rim was flush with the soil surface. Vials contained 50 mL of a 50% propylene glycol (*propane*-1, 2-diol) solution. At each site, five pitfall traps were placed in a regular arrangement in the central 10 m × 10 m area of each plot and marked with a flag. Four traps were placed in a square configuration, 5 m apart from each other, and the fifth was placed centrally. Pitfall traps were left for seven days before collection. Once collected, vials were then transported to the laboratory where the contents were transferred to a vial containing 70% ethanol. Sampling dates for pitfalls are scored as the day they were collected from the field.

In 2009, all plots were vacuum sampled prior to the bare earth application (PreBE), and again at 0, 3, 7, 14 and 28 days after treatment (DAT). Pitfall traps were used in all plots at PreBE at Rossbridge 2009, while at Inverleigh 2009 at PreBE, pitfall traps were only used in the broad-spectrum and untreated control plots. At both sites, pitfall traps were used across all plots at 0, 14 and 35 DAT. In 2010, there was no “bare earth” pesticide applied at the Inverleigh or Rossbridge sites. At these sites, all plots were vacuum sampled immediately after sowing (post sowing), and again at 0, 7, 14 and 28 DAT. Pitfall sampling occurred post sowing and at zero, 14 and 35 DAT.

### 2.4. Plant Assessments

In the 2009 trials, plant damage and plant density assessments were taken across all plots at 0, 3, 14 and 28 DAT. These assessments were only recorded at 28 DAT in the weed cover plots because the weeds were too dense to accurately assess plant numbers and plant damage at other sampling dates. Assessments were made in four random locations within each plot. At each sample location, a 0.5 m^2^ quadrat was placed on the ground along a row of plants, and the total number of canola plants within the quadrat was recorded. Overall plant damage was also assessed within the quadrat using a 0–10 scale, where 0 indicates no visible damage, 5 indicates 50% of the plants damaged and 10 indicates all plants dead or dying. This score has been used and validated in numerous studies involving earth mite pests [19,38,39]. In 2010, plant damage and plant density assessments were undertaken at 0, 7, 14 and 28 DAT. These were taken in 10 random locations within each plot. At each sample location, a wooden stick (the length equivalent to a row size of 0.25 m^2^ depending on row spacing) was placed on the ground along a row of plants, and the total number of wheat plants counted. Overall, plant damage was also assessed along rows marked by the stick as described above.

Yield estimates were undertaken at each site using a small plot harvester. In each plot, three strips were harvested the length of the plot and the average grain weight (t/ha) was recorded. Yield estimates were measured at Inverleigh and Rossbridge at 160 and 170 DAT, respectively in 2009, and at 224 and 218 DAT, respectively, in 2010.

### 2.5. Statistical Analysis

For all invertebrate and plant assessments, we calculated an average value for each plot. Plant density and invertebrates collected in the vacuum samples were converted to number of individuals of a taxon per m^2^. Data for some non-target invertebrates were combined into functional groups. These groups included predatory mites (Astigmata, Bdellidae, Mesostigmata) and predatory beetles (Anthicidae, Carabidae, Staphylinidae, Coccinellidae). Before analysis, all data were checked for normality using the Kolomogorov-Smirnov test (normal distribution) and Levene’s test (homogeneity of variances) following Sokal and Rohlf [40]. Where necessary, invertebrate numbers and plant density were log transformed (log(n + 1)) and feeding damage scores were arcsine square root transformed to achieve normality. However, to maintain biological meaning, all figures display untransformed data.

Overall effects of treatment on invertebrate numbers, plant density and plant damage were assessed using repeated measures analysis of variance (ANOVA). These were conducted for each individual invertebrate species/group for the field trials with sufficient numbers. For the invertebrate data, the PreBE or post sowing data was used as a covariate in the analyses. However, this was excluded in the pitfall data for Inverleigh 2009 given that no samples were undertaken at PreBE in most plots. For plant density and plant damage scores, the weed cover treatment was excluded from analyses in the 2009 trials given they were not assessed at most sampling dates.

A one-way analysis of covariance was performed for each invertebrate species/group to calculate studentized residuals, with PreBE or post sowing data included as a covariate. These studentized residuals were then used in analyses to assess differences between treatments at individual sampling dates for each trial. These were assessed using one-way ANOVAs with Tukey’s-*b* *post hoc* tests. For cases where repeated measures ANOVAs indicated significant effects for non-target invertebrates (see Table 2), we also calculated cumulative numbers of individuals from post-treatment sampling dates. At each field site, an average cumulative number was calculated per treatment, and one-way ANOVAs with Tukey’s-*b* *post hoc* tests were performed to investigate treatment differences. For plant density, plant damage and yield data, we conducted one-way ANOVAs with Tukey’s-*b* *post hoc* tests at individual sampling dates for each trial. We have not reported the one-way ANOVA outputs but the *post hoc* results are discussed and displayed in the figures and tables.

Finally, we estimated the relative reduction of invertebrate numbers following chemical treatment. For each individual invertebrate species (or group), we calculated the average number of individuals from all post-treatment sampling dates per plot. At each field site, a treatment average was estimated, and then divided by the average number of invertebrates from the untreated control plots at this site. This resulted in an estimate of the percentage reduction in the species/group relative to the control treatment (or increase as reflected by negative values). The weed cover treatment was excluded from this analysis because we were interested in assessing different chemical applications within the FCE framework.

Analyses were conducted in SPSS Statistics (version 20.0, IBM, New York, NY, USA).

## 3. Results

### 3.1. Invertebrates

A large number of pests and non-target invertebrates were collected from all four field trials, with the abundance of species and groups varying greatly with collection method. Several groups were not analyzed given low and/or inconsistent numbers collected across the field trials (e.g., spiders were counted within every sample but only present in numbers greater than an average of 1/m^2^ at Rossbridge in 2010). For the purpose of this study, we were particularly interested in the major pests present: *H. destructor* and *Penthaleus* species*.* Data for non-target invertebrates were combined into functional groups. These groups included predatory mites (Astigmata, Bdellidae, Mesostigmata) and predatory beetles (Anthicidae, Carabidae, Staphylinidae). We also analyzed Collembola (Hypogsdtruridae), Oribatidae and Formicidae. Based on trapping efficiency [32,36], vacuum data was used for all groups, except for the Formicidae and predatory beetles for which pitfall data was used.

#### 3.1.1. Pest Species

**Figure 2:**
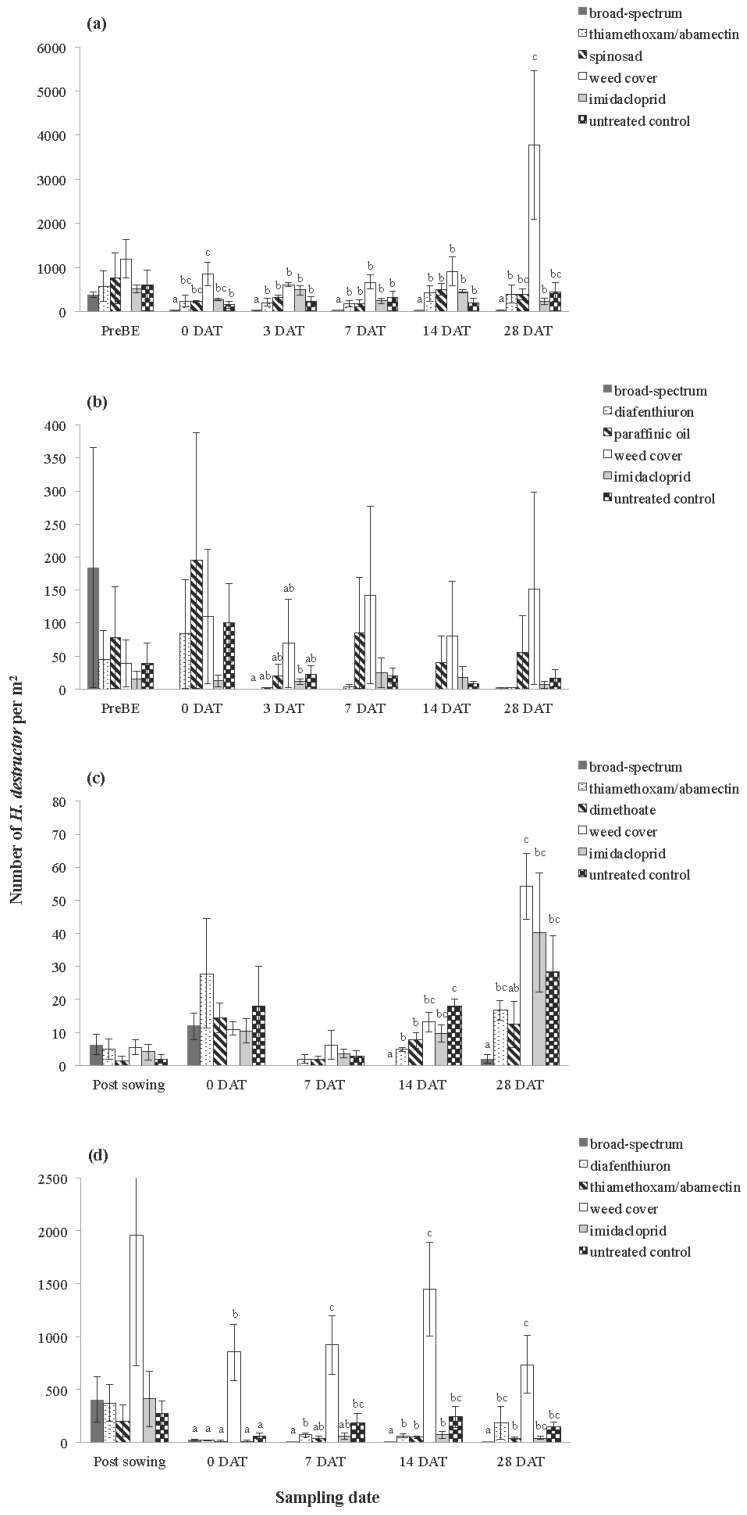
Average number of *H. destructor* collected using vacuum sampling at (**a**) Inverleigh 2009, (**b**) Rossbridge 2009, (**c**) Inverleigh 2010 and (**d**) Rossbridge 2010. Error bars represent standard errors of the mean. Different letters above bars indicate significantly different means at each sampling date (at the *p* < 0.05 level, Tukey’s-*b* *post hoc* test).

Treatments had an overall impact on *H. destructor* numbers across the majority of trials (Table 2). Significant treatment effects were present at all sites at most sampling dates for *H. destructor*, except for Rossbridge 2009 where differences were only present at three DAT (Figure 2). The weed cover treatments tended to have the highest mite numbers across trials. The broad-spectrum pesticides had significantly fewer *H. destructor* than the other treatments at most trials, although there were some exceptions. There was no difference between chemical treatments at most sampling dates at the Rossbridge 2009 site (Figure 2b) and for the early sampling dates at the Rossbridge 2010 site (Figure 2d). Furthermore, no differences were evident between the broad-spectrum pesticides and other treatments at the early sampling dates at Inverleigh 2010 (Figure 2c). In most trials, there were no significant differences detected between the selective pesticides, and these did not generally differ from the untreated controls. Diafenthiuron, dimethoate and thiamethoxam/abamectin typically reduced *H. destructor* numbers by 44% to 92%, but patterns were not consistent across sampling dates and trials.

**Table 2:** Repeated measures ANOVAs comparing the overall treatment effects on pest species and non-target invertebrate numbers collected from vacuum (V) and pitfall (P) samples across each field site.

Trial Site	Functional Group	Sampling Type	df	MS	*F*-value	*p*
Inverleigh 2009	*H. destructor*	V	5, 18	9.030	32.489	<0.001
	*Penthaleus* spp.	V	5, 18	4.954	13.104	<0.001
	Collembola	V	5, 18	0.009	0.181	0.966
	Predatory mites	V	5, 18	1.317	7.153	0.001
	Formicidae	P	5, 18	0.379	5.592	0.003
	Predatory beetles	P	5, 18	4.035	4.313	0.009
						
Rossbridge 2009	*H. destructor*	V	5, 18	2.883	2.520	0.070
	*Penthaleus* spp.	V	5, 18	8.927	9.301	<0.001
	Collembola	V	5, 18	2.248	17.565	<0.001
	Oribatidae	V	5, 18	2.137	1.616	0.209
	Predatory mites	V	5, 18	1.536	4.613	0.008
	Formicidae	P	5, 18	0.400	5.425	0.003
	Predatory beetles	P	5, 18	4.262	5.498	0.003
						
Inverleigh 2010	*H. destructor*	V	5, 18	1.175	4.920	0.006
	*Penthaleus* spp.	V	5, 18	2.275	13.948	<0.001
	Collembola	V	5, 18	0.682	4.503	0.009
	Oribatidae	V	5, 18	0.991	0.181	0.966
	Predatory mites	V	5, 18	0.165	0.405	0.839
	Formicidae	P	5, 18	0.169	2.962	0.042
	Predatory beetles	P	5, 18	2.437	1.770	0.170
						
Rossbridge 2010	*H. destructor*	V	5, 18	8.347	22.831	<0.001
	*Penthaleus* spp.	V	5, 18	1.123	2.324	0.088
	Collembola	V	5, 18	0.666	26.237	<0.001
	Oribatidae	V	5, 18	0.316	0.627	0.681
	Predatory mites	V	5, 18	1.808	20.471	<0.001
	Formicidae	P	5, 18	0.056	3.858	0.016
	Predatory beetles	P	5, 18	0.797	0.722	0.616

**Figure 3:**
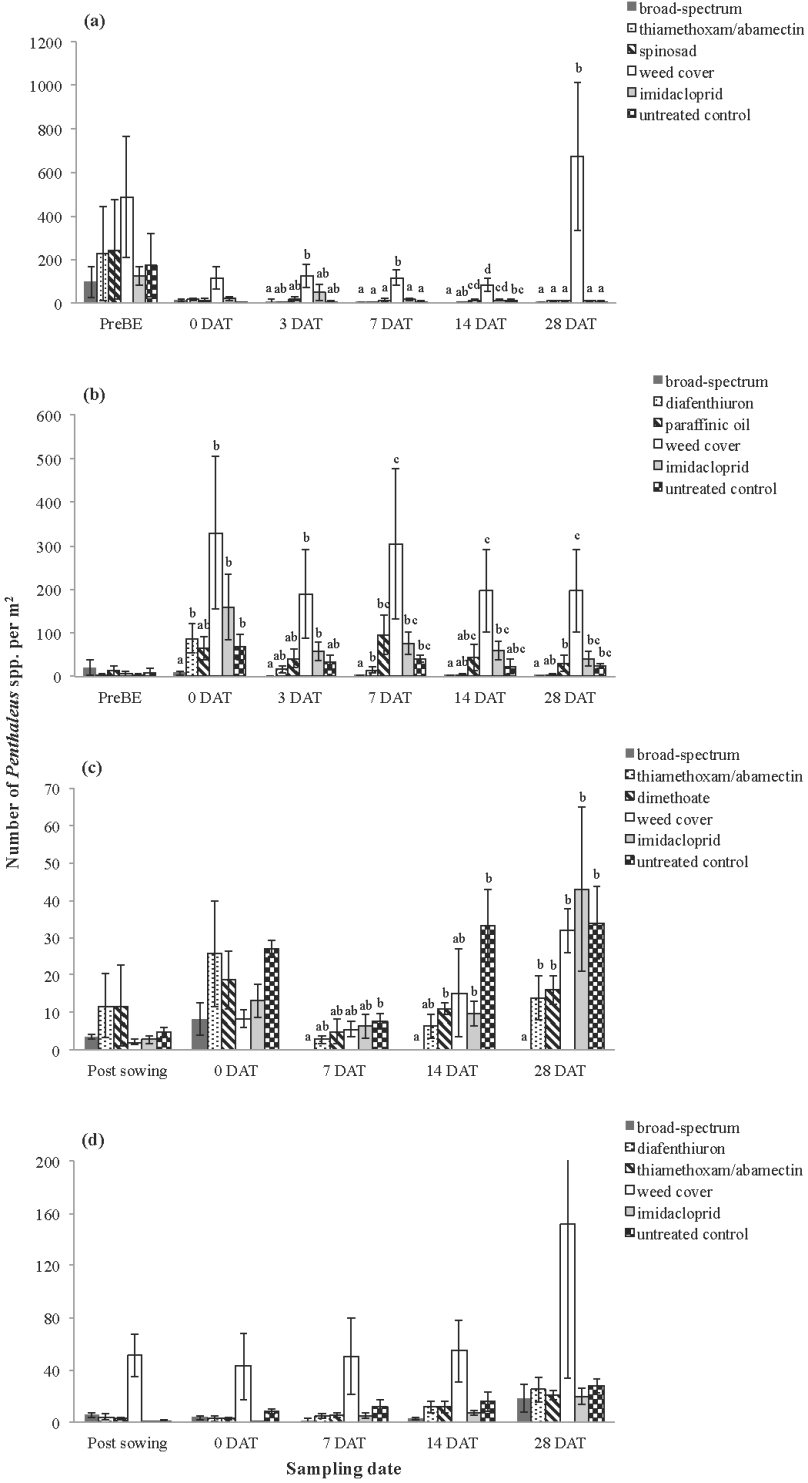
Average number of *Penthaleus* spp. collected using vacuum sampling at (**a**) Inverleigh 2009, (**b**) Rossbridge 2009, (**c**) Inverleigh 2010 and (**d**) Rossbridge 2010. Error bars represent standard errors of the mean. Different letters above bars indicate significantly different means at each sampling date (at the *p* < 0.05 level, Tukey’s-*b* post hoc test).

For *Penthaleus* spp., the repeated measures ANOVAs showed a significant treatment effect at three of the four trials (Table 2). Significant treatment effects were observed at most sampling dates for all sites, except Rossbridge 2010 (Figure 3). Where treatments were significant, the broad-spectrum treatments had fewer *Penthaleus* spp. than the weed cover treatments. At Rossbridge 2009 and Inverleigh 2010, significantly fewer *Penthaleus* spp. were present within the broad-spectrum treatments compared with the imidacloprid and untreated control treatments. The same pattern was observed at Inverleigh 2009 at 14 DAT but not for the other sampling dates at this site. As observed for *H. destructor*, some selective pesticides tended to reduce *Penthaleus* spp. numbers by 25% to 69%, but these patterns were not consistent across sampling dates and trials, and, in many cases, not significantly different to the untreated control plots (Figure 3).

#### 3.1.2. Non-Target Invertebrates

Collembola were present in large numbers at all sites, and treatment had an overall significant effect at Rossbridge 2009, Inverleigh 2010 and Rossbridge 2010 (Table 2). The broad-spectrum treatments and the diafenthiuron treatment typically reduced Collembola numbers compared with the other treatments (Table 3). At individual sampling dates, significant treatment effects were present at all sites for at least two sampling dates (Supplementary Table S1). The untreated controls had similar numbers of Collembola as the weed cover, imidacloprid, paraffinic oil and dimethoate treatments (irrespective of sampling date).

Similar to Collembola, predatory mites were present in sufficient numbers at all sites. Significant treatment effects were found at Inverleigh 2009, Rossbridge 2009 and Rossbridge 2010 (Table 2). The broad-spectrum treatments tended to have lower mite numbers compared with the weed cover, imidacloprid and untreated control treatments (Table 3). At Rossbridge 2010, the weed cover treatment had significantly more predatory mites compared to the other treatments at most sampling dates, with an overall increase of almost 400% compared to the controls (Supplementary Table S1). However, this was not consistent with the other sites, particularly Inverleigh 2009 where the weed treatment tended to have fewer predatory mites. At sites where treatment effects were significant, the broad-spectrums typically had fewer predatory mites than the untreated controls (0%–63% reduction).

Oribatidae were present in sufficient numbers in three sites (Rossbridge 2009 and 2010, and Inverleigh 2010); treatment had no overall impact on numbers in any of these trials (Table 2). However, significant treatment effects were present at 28 DAT at Rossbridge 2009, where the diafenthiuron treatment had significantly lower numbers of Oribatidae than the paraffinic oil and untreated control treatments (Supplementary Table S1), involving a reduction of 71%. For Formicidae, treatments had an overall impact on numbers at all sites (Table 2). Significant effects were present at individual sampling dates at each site (Supplementary Table S2). In most cases, the broad-spectrum treatments had significantly fewer Formicidae than the untreated control (22%–81% reduction), and to a lesser extent the imidacloprid treatment (0%–23% reduction). The weed cover treatment also had significantly fewer Formicidae than the untreated control and imidacloprid treatments at several dates across these trials. 

Predatory beetles were found across all sites, and overall treatment effects were seen at Inverleigh 2009 and Rossbridge 2009 (Table 2); however, there were relatively few significant treatment effects at individual sampling dates (Supplementary Table S2). For the 2009 sites, the imidacloprid treatment had significantly more predatory beetles than the weed cover treatment at zero DAT. The broad-spectrum treatments had significantly fewer beetles than the imidacloprid treatment at zero DAT at Rossbridge 2009, and at 35 DAT at Inverleigh 2009. This treatment also had significantly fewer beetles than the dimethoate treatment at 14 DAT at Inverleigh 2010 (Supplementary Table S2), with reductions ranging from 31% to 48%. When considering cumulative numbers, the broad-spectrum treatment had significantly fewer beetles than the imidacloprid treatment at a single site (Table 3).

**Table 3:** Pre-treatment and cumulative numbers of non-target invertebrates (and standard errors) collected from all sampling dates post-chemical treatment for each case where repeated measures ANOVAs indicated a significant effect. Results from one-way ANOVAs comparing all treatments across each field site are displayed, where PreBE or post sowing sampling dates were used as covariates in the analyses. Different letters indicate significantly different means (at the *p* < 0.05 level, Tukey’s-*b* *post hoc* test).

Functional Group	Trial Site	Treatment	Pre-treatment ± se *	Post-treatment ± se (Cumulative)	*Post hoc* Tests
Collembola	Rossbridge 2009	Broad-spectrum	709 ± 254	1910 ± 654	a
		Diafenthiuron	545 ± 270	2471 ± 157	a
		Paraffinic oil	521 ± 160	5600 ± 772	b
		Weed cover	523 ± 190	8867 ± 2395	b
		Imidacloprid	586 ± 204	7632 ± 1768	b
		Untreated control	512 ± 148	6406 ± 1835	b
	Inverleigh 2010	Broad-spectrum	6367 ± 1789	28303 ± 2625	
		Thiamethoxam/abamectin	8985 ± 2939	28538 ± 8827	
		Dimethoate	9823 ± 2506	46174 ± 3125	
		Weed cover	6976 ± 2354	47028 ± 8540	
		Imidacloprid	10509 ± 1729	50818 ± 4650	
		Untreated control	14511 ± 2501	49211 ± 6949	
	Rossbridge 2010	Broad-spectrum	3557 ± 1777	37035 ± 3466	a
		Diafenthiuron	1679 ± 541	45098 ± 3932	a
		Thiamethoxam/abamectin	1555 ± 655	49813 ± 3796	a
		Weed cover	1264 ± 217	145550 ± 6794	c
		Imidacloprid	3293 ± 1513	68824 ± 6279	b
		Untreated control	833 ± 100	82464 ± 5788	b
Predatory mites	Inverleigh 2009	Broad-spectrum	198 ± 54	1073 ± 193	
		Thiamethoxam/abamectin	156 ± 32	1277 ± 336	
		Spinosad	119 ± 24	1241 ± 134	
		Weed cover	140 ± 40	545 ± 154	
		Imidacloprid	123 ± 34	1331 ± 51	
		Untreated control	181 ± 52	1681 ± 298	
	Rossbridge 2009	Broad-spectrum	77 ± 19	73 ± 22	a
		Diafenthiuron	76 ± 19	117 ± 30	ab
		Paraffinic oil	62 ± 26	192 ± 37	ab
		Weed cover	120 ± 37	216 ± 48	b
		Imidacloprid	115 ± 38	282 ± 57	b
		Untreated control	141 ± 41	294 ± 63	b
	Rossbridge 2010	Broad-spectrum	519 ± 196	249 ± 17	a
		Diafenthiuron	300 ± 148	417 ± 74	b
		Thiamethoxam/abamectin	732 ± 308	376 ± 103	b
		Weed cover	548 ± 185	2202 ± 214	b
		Imidacloprid	601 ± 108	332 ± 53	b
		Untreated control	338 ± 192	491 ± 30	b
Formicidae	Inverleigh 2009	Broad-spectrum	-	8 ± 2	a
		Thiamethoxam/abamectin	-	24 ± 4	b
		Spinosad	-	25 ± 6	b
		Weed cover	-	10 ± 3	ab
		Imidacloprid	-	24 ± 5	b
		Untreated control	-	25 ± 7	b
	Rossbridge 2009	Broad-spectrum	2 ± 0	4 ± 0	
		Diafenthiuron	7 ± 1	13 ± 1	
		Paraffinic oil	8 ± 2	14 ± 3	
		Weed cover	3 ± 1	6 ± 1	
		Imidacloprid	6 ± 2	15 ± 4	
		Untreated control	10 ± 2	19 ± 4	
	Inverleigh 2010	Broad-spectrum	25 ± 8	9 ± 2	
		Thiamethoxam/abamectin	18 ± 2	8 ± 2	
		Dimethoate	17 ± 2	15 ± 3	
		Weed cover	28 ± 2	13 ± 3	
		Imidacloprid	21 ± 3	15 ± 3	
		Untreated control	10 ± 2	11 ± 3	
	Rossbridge 2010	Broad-spectrum	15 ± 2	4 ± 1	a
		Diafenthiuron	8 ± 2	6 ± 1	ab
		Thiamethoxam/abamectin	9 ± 3	5 ± 1	ab
		Weed cover	10 ± 2	4 ± 1	ab
		Imidacloprid	12 ± 3	7 ± 0	b
		Untreated control	16 ± 3	7 ± 1	ab
Predatory beetles	Inverleigh 2009	Broad-spectrum	-	7 ± 1	a
		Diafenthiuron	-	6 ± 1	ab
		Paraffinic oil	-	9 ± 1	ab
		Weed cover	-	4 ± 1	ab
		Imidacloprid	-	12 ± 2	b
		Untreated control	-	10 ± 3	ab
	Rossbridge 2009	Broad-spectrum	1 ± 0	3 ± 1	
		Thiamethoxam/abamectin	1 ± 0	10 ± 3	
		Dimethoate	1 ± 0	7 ± 2	
		Weed cover	1 ± 0	3 ± 1	
		Imidacloprid	1 ± 0	7 ± 2	
		Untreated control	1 ± 0	7 ± 2	

* No pre-treatment data for Formicidae or predatory beetles at Inverleigh 2009 as no pitfall samples were taken at this time point.

**Figure 4:**
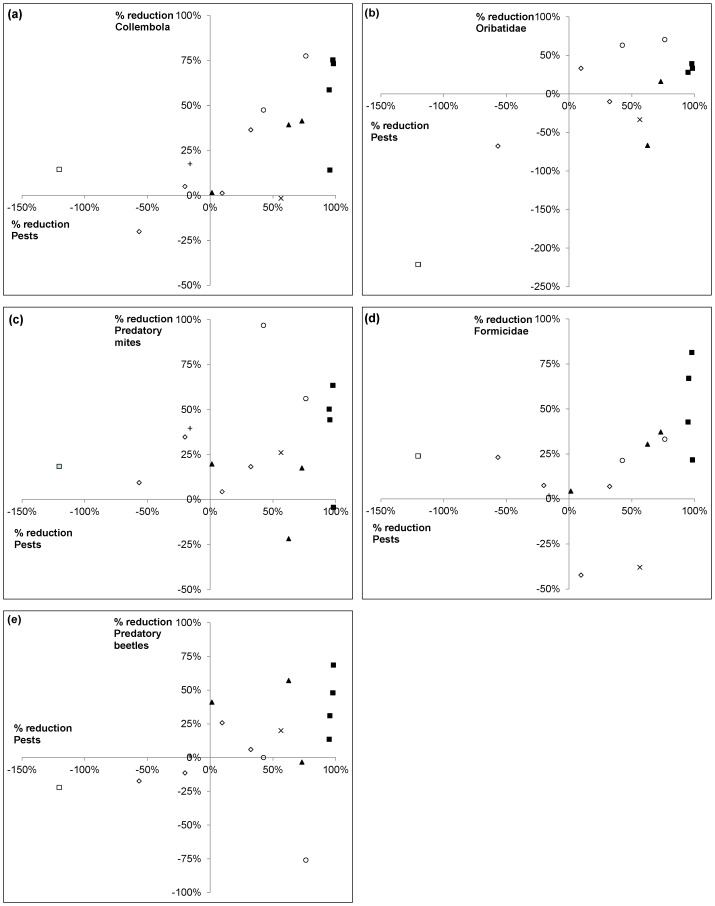
Percentage reduction of pest numbers (*H. destructor* and *Penthaleus* spp.) relative to reductions in (**a**) Collembola, (**b**) Oribatidae, (**c**) Predatory mites, (**d**) Formicidae and (**e**) Predatory beetles after exposure to broad-spectrums (■), diafenthiuron (○), thiamethoxam/abamectin (▲), dimethoate (×), imidacloprid (◇), spinosad (+) and paraffinic oil (□).

Figure 4 clearly depicts the reduction in non-target invertebrates relative to the reduction in pest numbers after exposure to chemical treatments applied across the four trials. For most treatments, reductions were seen in both the pest and non-target groups. There were only a few instances where pest numbers (*H. destructor* and *Penthaleus* spp. combined) were reduced and the non-target group was only marginally reduced, or not reduced at all. The application of broad-spectrum chemicals typically resulted in substantial reductions in pest numbers, but, at the same time, these treatments negatively impacted numbers of Collembola, Oribatidae, predatory mites, Formicidae and predatory beetles (Figure 4). The exception was at Inverleigh 2010, where the conventional treatment caused no detectable reduction in predatory mite numbers (Figure 4c). Although trends varied across groups, the thiamethoxam/abamectin treatment generally showed limited or no reductions to non-target invertebrates, while reducing pest numbers by >65% in two of the three trial sites. Diafenthiuron caused substantial reductions in Collembola, Oribatidae and predatory mite numbers but did not negatively impact predatory beetles (Figure 4).

### 3.2. Plant Assessments

**Figure 5:**
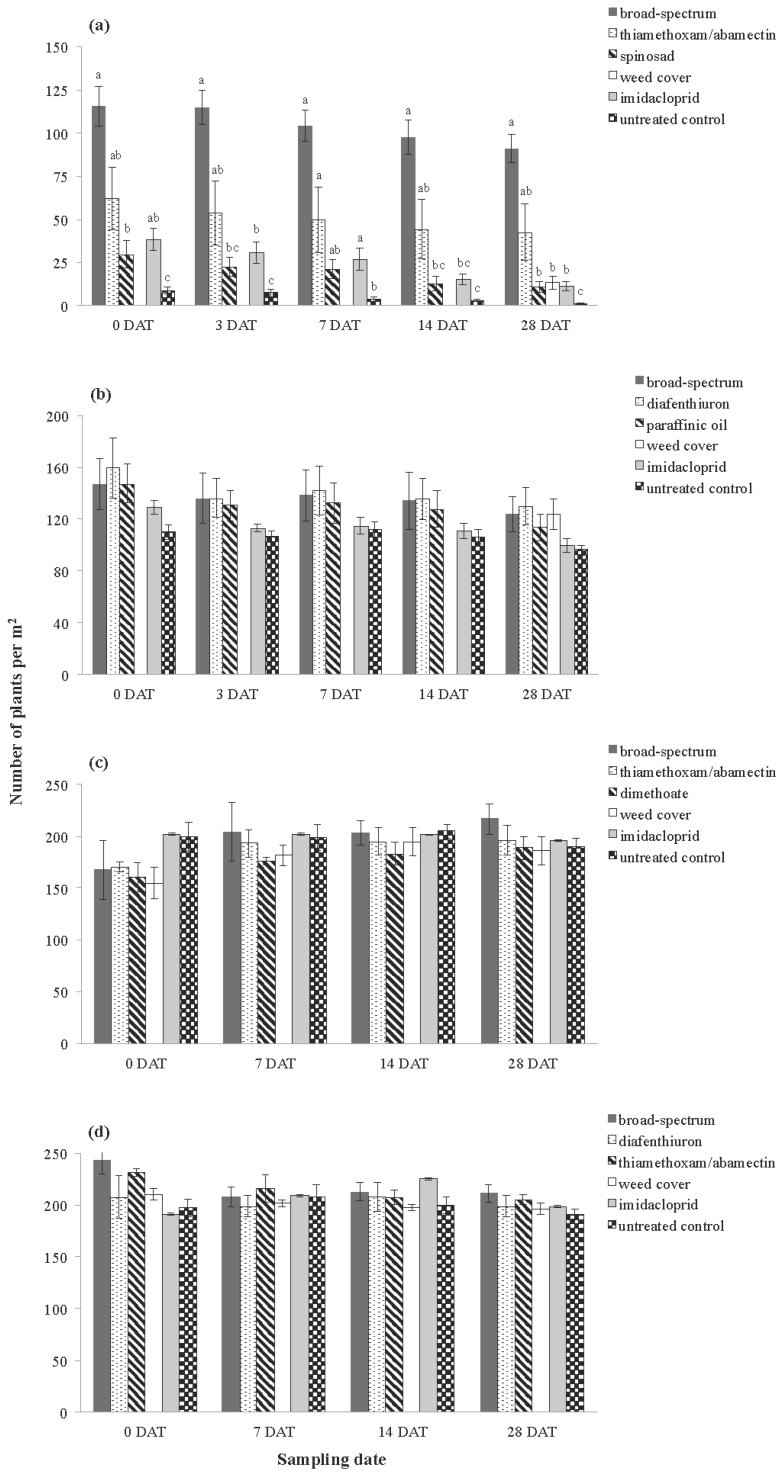
Average number of plants at (**a**) Inverleigh 2009, (**b**) Rossbridge 2009, (**c**) Inverleigh 2010 and (**d**) Rossbridge 2010. Plant counts were not scored for the Weed cover treatment at zero, three, seven and 14 DAT at Inverleigh 2009 and Rossbridge 2009. Error bars represent standard errors of the mean. Different letters above bars indicate significantly different means at each sampling date (at the *p* < 0.05 level, Tukey’s-*b* *post hoc* test).

With the exception of Inverleigh 2009, there were few treatment effects on plant density across the four trials (Figure 5). Treatment had an overall impact on the number of plants at Inverleigh 2009 (Table 4), and treatment effects were evident at all sampling dates (Figure 5a). At this site, the broad-spectrum treatment had the highest number of plants at all sampling dates, followed by the thiamethoxam/abamectin treatment. Spinosad and the imidacloprid treatments had higher plant numbers compared with the untreated controls, although these were not significantly different at any sampling date. The number of plants present within the weed cover treatment could only be scored at 28 DAT; and this treatment had significantly fewer plants (85% change) than the broad-spectrum treatment but significantly more plants (93% change) than the untreated control (Figure 5a).

**Figure 6:**
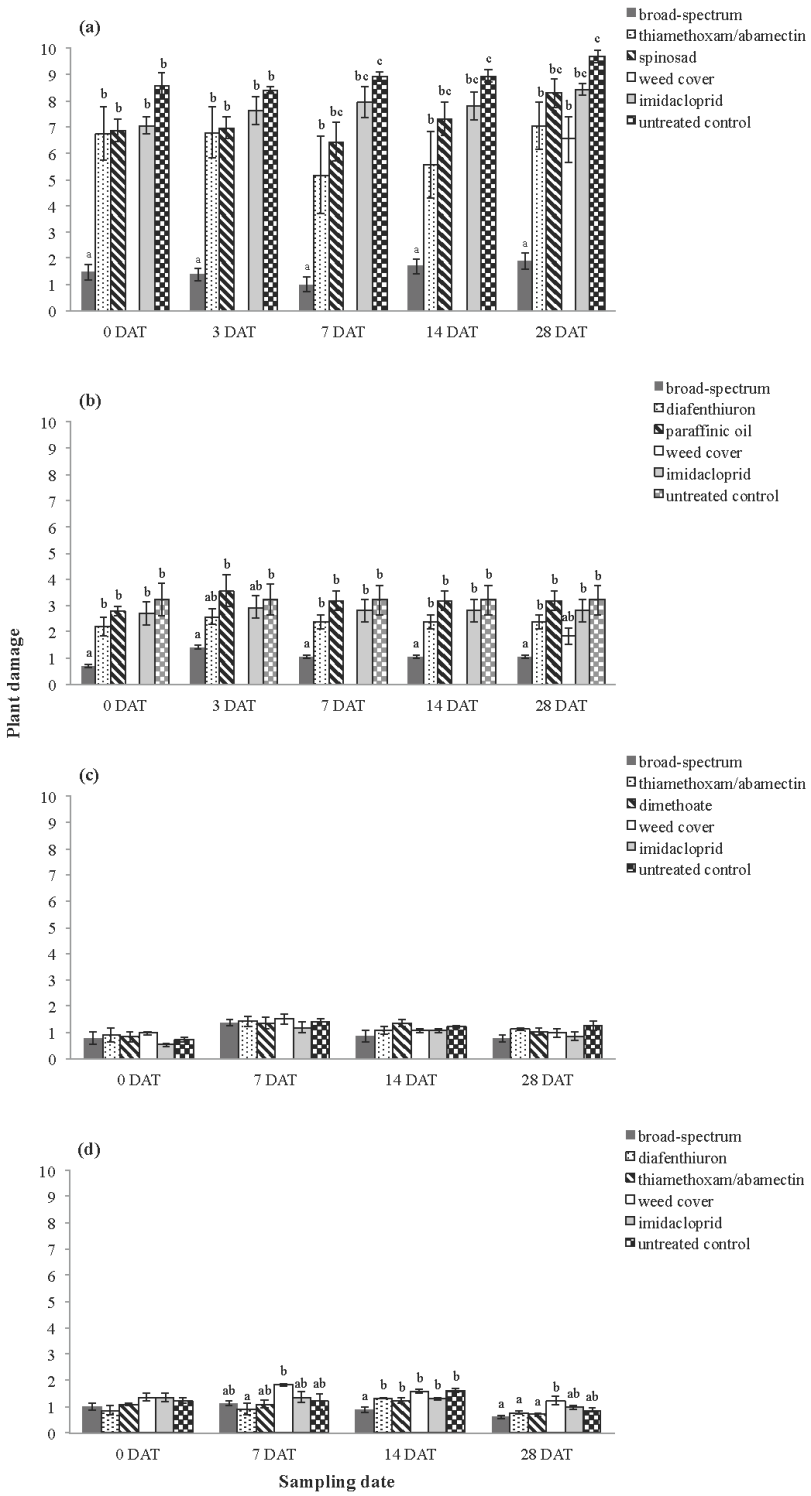
Average plant damage scores at (**a**) Inverleigh 2009, (**b**) Rossbridge 2009, (**c**) Inverleigh 2010 and (**d**) Rossbridge 2010. Plant damage was not scored for the Weed cover treatment at zero, three, seven and 14 DAT at Inverleigh 2009 and Rossbridge 2009. Error bars represent standard error of the mean. Different letters above bars indicate significantly different means at each sampling date (at the *p* < 0.05 level, Tukey’s-*b* *post hoc* test).

There were overall treatment effects for plant damage scores at all sites except for Inverleigh 2010 (Table 4). The feeding damage sustained to wheat (at Inverleigh and Rossbridge 2010) was considerably lower than the damage to canola (at Inverleigh 2009 and Rossbridge 2009). Plots treated with the broad-spectrum pesticides tended to have lower plant damage scores than all other treatments; these effects were significantly different at several sampling dates across most trials (Figure 6). The untreated controls tended to suffer the highest level of feeding damage. The amount of damage in the weed cover treatment was lower than the untreated controls at Inverleigh 2009 and Rossbridge 2009 (assessed at 28 DAT). The thiamethoxam/abamectin treatment had lower plant damage scores than the untreated control, although this difference was not always significant. The levels of plant damage among the remaining selective treatments were variable across the trials (Figure 6).

For crop yield, significant treatment effects were evident at the canola sites (Inverleigh 2009, Rossbridge 2009) but not the two wheat sites (Inverleigh 2010, Rossbridge 2010) (Table 5). At Inverleigh 2009, the broad-spectrum treatment had significantly higher yield than the weed cover, imidacloprid and untreated control treatments. The untreated control also had significantly lower yield than the thiamethoxam/abamectin treatment, but no other differences were detected between the selective chemicals. Yield was 91% lower in the untreated control compared to the conventional treatment, while yield in the selective chemical treatments varied from 27% to 64% that of the conventional treatment. At Rossbridge 2009, the untreated control and the weed cover treatments had the lowest yields, although the only significant difference was between diafenthiuron and the weed cover treatment (Table 5), where there was a 53% reduction in yield.

**Table 4:** Repeated measures ANOVAs comparing the overall treatment effects for number of plants and plant damage scores across each field site.

Trial Site	Plant Measure	df	MS	*F*-Value	*p*
Inverleigh 2009	Number of plants	5, 18	4.991	9.247	0.001
**	Plant damage	5, 18	2.177	21.171	<0.001
Rossbridge 2009	Number of plants	5, 18	0.045	1.277	0.322
**	Plant damage	5, 18	0.252	6.979	0.002
Inverleigh 2010	Number of plants	5, 18	0.009	0.896	0.505
**	Plant damage	5, 18	0.006	1.339	0.293
Rossbridge 2010	Number of plants	5, 18	0.004	1.210	0.344
	Plant damage	5, 18	0.020	9.529	<0.001

**Table 5:** Average yield estimates (and standard errors) at each trial site. Different letters indicate significant differences (at the *p* < 0.05 level, Tukey’s-*b* *post hoc* test).

Trial Site	Treatment	Yield (t/ha) ± se	*Post hoc* Tests
Inverleigh 2009	Bifenthrin and omethoate	2.04 ± 0.19	a
	Thiamethoxam/abamectin	1.48 ± 0.47	ab
	Spinosad	1.07 ± 0.35	abc
	Weed cover	0.39 ± 0.17	bc
	Imidacloprid	0.74 ± 0.18	bc
	Untreated control	0.18 ± 0.11	c
			
Rossbridge 2009	Bifenthrin and omethoate	1.37 ± 0.07	ab
	Diafenthiuron	1.43 ± 0.28	a
	Paraffinic oil	1.25 ± 0.36	ab
	Weed cover	0.67 ± 0.31	b
	Imidacloprid	1.33 ± 0.42	ab
	Untreated control	0.99 ± 0.57	ab
			
Inverleigh 2010	Bifenthrin	5.20 ± 0.32	
	Thiamethoxam/abamectin	4.23 ± 0.76	
	Dimethoate	4.52 ± 0.71	
	Weed cover	4.19 ± 0.98	
	Imidacloprid	4.80 ± 0.53	
	Untreated control	4.61 ± 0.97	
			
Rossbridge 2010	Bifenthrin	4.82 ± 0.20	
	Diafenthiuron	4.71 ± 0.34	
	Thiamethoxam/abamectin	4.90 ± 0.15	
	Weed cover	4.93 ± 0.17	
	Imidacloprid	5.04 ± 0.40	
	Untreated control	4.91 ± 0.22	

## 4. Discussion

The FCE framework put forward here provides a method to assess the likely IPM suitability of new chemical compounds in the field. Although the selective pesticides tested in this study were not as effective on mite pests as the broad-spectrum products, several chemicals did provide a moderate level of control (*i.e.*, ~55%–75% reductions in pest numbers) and some had very few (if any) negative effects on non-target invertebrates. Using field trials within this context, we have previously shown that the impact of broad-spectrum chemicals varies between groups of non-target organisms and is not easily predictable from standardized tests [33]. While overall IOBC ratings can help predict changes in invertebrate communities within broadacre systems when considered across years [28], the results of laboratory assays on individual chemicals do not necessarily extrapolate to field conditions [34]. This may reflect a number of factors like exposure levels in a complex landscape, inherent levels of resistance in species that have not directly been tested and tolerant life stages of non-target species that can overestimate the likely impacts of agricultural chemicals [41,42,43]. This FCE framework is one way of assessing non-target effects directly relevant to field conditions.

Our work provides yet another example where reductions in pest numbers within a field context do not always correspond with increased crop yields [2]. While the broad-spectrum treatments in our trials typically had the highest yield and lowest plant damage scores, significant differences were only evident in two trials. Even then, there were no marked yield differences between the broad-spectrum pesticides and several of the selective treatments including thiamethoxam/abamectin, diafenthiuron and dimethoate. Because reductions in yield due to earth mites are only likely under high pest pressures when present at susceptible stages of plant development [38], a useful advancement to the FCE framework would be to add a third axis to Figure 1 to indicate pest pressure; when pest pressure is low, any yield effects would be predicted to be minor, whereas when pest pressure is high, a large impact on yield might be expected particularly if there is no plant compensation at later stages of crop development [38].

Based on the results presented here, further exploration of a number of products (either as new registrations into grains, or in the case of dimethoate at reduced field rates) is warranted. Some chemicals, such as diafenthiuron and thiamethoxam/abamectin, showed promise in combatting mite pests with reduced non-target effects, although the levels of efficacy differed across field trials. Other chemicals, such as spinosad and paraffinic oil, showed little promise in these trials, and future research into these pesticides is probably not warranted. The broad-spectrum pesticides provided the greatest efficacy against *H. destructor* and *Penthaleus* species and, at this stage, are likely to remain the only main chemical options at times of high pest pressure. Within an IPM context, spray decisions should not solely focus on the which chemical to apply; they should consider spray timing and be informed through economic pest thresholds (where available) that aim to reduce pest numbers and maintain them at populations below those causing economic injury [44,45]. In the case of *H. destructor*, attempts have been made to devise economic thresholds in Australian cropping systems, although these have proved problematic [38,46].

As displayed in the FCE framework, the use of pesticide seed treatments in our trials was effective when low-moderate mite numbers were present, but less so in situations with high pest pressures. With the exception of Inverleigh 2009, the use of imidacloprid as a seed treatment resulted in relatively low plant damage scores, and plant densities of a similar level to plots treated with broad-spectrum pesticides. There were also no significant reductions in crop yield in plots treated with imidacloprid compared with the broad-spectrum treatments. Importantly, there were very few mortality effects observed for non-target groups in the imidacloprid plots across each of the four trials, consistent with other studies on this chemical when used as a seed treatment [47]. These results further demonstrate the value that pesticide seed dressings can have in Australian dryland systems, particularly when pest pressures are not high (see also Macfadyen *et al.* [2]), although environmental concerns remain around the use of neonicotinoid chemicals [48]. Similar to the inclusion of the imidacloprid treatment in each trial, we included a weed cover treatment across all trial sites. We were interested to explore the possibility of providing an alternative food source for pests during the susceptible seedling stage of both canola and wheat. Despite earth mites commonly attacking the weed species found within our trial plots (e.g., capeweed, clover, rye grass) [17,19,49], we found little economic benefit of this approach.

## 5. Conclusions

This work highlights the challenges in unraveling the relationships between pesticide, target pest, non-targets, and overall plant effects. Further work is required to tease apart the impacts of chemicals on non-target beneficials and to accurately assess the efficacy of selective chemicals. This is especially the case since we focused largely on the short-term impacts of pesticides, and as a result, sublethal impacts were not considered in depth [50,51]. Nonetheless, our results show that broad-spectrum pesticides currently provide the greatest efficacy against key grain pests but typically have greater impacts on non-target invertebrates. There is value in using a field-based framework to assess IPM compatibility like the one proposed in this study. In agro-ecosystems where data is available, this framework could be extended to consider beneficial invertebrates as a whole, rather than assessing individual groups as we have done here. This would require a detailed understanding of the pest suppression levels exerted by individual beneficial groups in order to apply appropriate weightings before combining into a single measure. Our findings demonstrate the opportunity for selective chemicals to be used more widely by Australian grain growers. In particular, the role of selective pesticides appears suited to wheat and/or when canola crops are sown with pesticide seed dressings. Growers therefore have the potential to improve sustainability and environmental performance without a reduction in productivity.

## Supplementary Files

Supplementary File 1
