# Supplementary material for: A Framework for Identifying Selective Chemical Applications for IPM in Dryland Agriculture
Source: Insects. 2015 Dec 16;6(4):988–1012. doi: 10.3390/insects6040988 (PMC4693183; doi:10.3390/insects6040988)
Supplement: Supplementary File 1 [file insects-06-00988-s001.pdf]

Supplementary Information

Table S1. Average number of non-target invertebrates collected from vacuum samples and results from one-way ANOVAs comparing treatments at each field site. PreBE or post sowing sampling dates were used as covariates in the analyses. Different letters indicate significantly different means at each sampling date (at the $p < 0.05$ level, Tukey's-*b post hoc* test).

Functional Group	Trial Site	Treatment	0-DAT	3-DAT	7-DAT	14-DAT	28-DAT
Collembola	Inverleigh 2009	Broad-spectrum	6041 [a]	2041	2614	4455	3238 [b]
		Thiamethoxam/abamectin	3463 [ab]	2674	2000	4730	4748 [b]
		Spinosad	7200 [a]	2494	1900	4646	2812 [b]
		Weed cover	2280 [b]	1892	2080	4878	11622 [a]
		Imidacloprid	4900 [ab]	2908	3221	4497	3042 [b]
		Untreated control	2762 [b]	2432	2537	4379	5039 [b]
	Rossbridge 2009	Broad-spectrum	624	190 [a]	42 [a]	411 [ab]	643 [a]
		Diafenthiuron	1299	177 [a]	144 [a]	92 [a]	759 [a]
		Paraffinic oil	1142	437 [ab]	1004 [b]	1042 [c]	1976 [b]
		Weed cover	1320	728 [b]	1411 [b]	933 [bc]	4475 [b]
		Imidacloprid	1376	467 [ab]	1044 [b]	829 [bc]	3916 [b]
		Untreated control	1194	331 [ab]	877 [b]	885 [bc]	3118 [b]
	Inverleigh 2010	Broad-spectrum	21833	-	2841	1610 [a]	2019 [a]
		Thiamethoxam/abamectin	13811	-	3761	6844 [ab]	4122 [ab]
		Dimethoate	21528	-	7291	9565 [b]	7790 [b]
		Weed cover	22683	-	6857	9391 [b]	8097 [b]
		Imidacloprid	26867	-	6097	8980 [b]	8875 [b]
		Untreated control	24956	-	6158	10147 [b]	7951 [b]
	Rossbridge 2010	Broad-spectrum	6572	-	17631 [a]	5427 [a]	7406 [ab]
		Diafenthiuron	6390	-	24088 [a]	9337 [a]	5283 [a]
		Thiamethoxam/abamectin	6598	-	21051 [a]	10379 [a]	11786 [bc]
		Weed cover	11833	-	52601 [b]	41318 [b]	39798 [e]
		Imidacloprid	7730	-	27604 [a]	16244 [c]	17246 [cd]
		Untreated control	8704	-	32118 [a]	12259 [a]	29383 [de]
Oribatidae	Rossbridge 2009	Broad-spectrum	9	33	85	107	35 [ab]
		Diafenthiuron	54	42	34	40	10 [a]
		Paraffinic oil	75	215	476	325	358 [b]
		Weed cover	310	99	261	92	67 [ab]
		Imidacloprid	237	85	327	90	215 [ab]
		Untreated control	156	50	178	74	125 [b]
	Inverleigh 2010	Broad-spectrum	11	-	3	0	3
		Thiamethoxam/abamectin	11	-	14	0	0
		Dimethoate	14	-	0	11	0
		Weed cover	17	-	0	0	0
		Imidacloprid	8	-	6	0	0
		Untreated control	11	-	6	0	3
	Rossbridge 2010	Broad-spectrum	44	-	97	184	178
		Diafenthiuron	22	-	77	65	93
		Thiamethoxam/abamectin	106	-	155	218	163
		Weed cover	176	-	272	349	422
		Imidacloprid	53	-	155	157	155
		Untreated control	32	-	205	176	258

Table S1. *Cont.*

Functional	Trial Site	Treatment	0-DAT	3-DAT	7-DAT	14-DAT	28-DAT
Predatory mites	Inverleigh 2009	Broad-spectrum	357	433 [a]	133 [ab]	143 [ab]	7 [a]
		Thiamethoxam/abamectin	244	466 [a]	277 [bc]	283 [ab]	7 [a]
		Spinosad	464	410 [a]	222 [bc]	144 [ab]	1 [a]
		Weed cover	355	53 [b]	52 [a]	84 [a]	2 [a]
		Imidacloprid	491	361 [a]	262 [bc]	216 [ab]	1 [a]
		Untreated control	394	437 [a]	445 [c]	340 [b]	65 [b]
	Rossbridge 2009	Broad-spectrum	1 [a]	22	24 [a]	22	5 [a]
		Diafenthiuron	30 [b]	21	49 [b]	12	5 [a]
		Paraffinic oil	30 [b]	16	81 [bc]	59	6 [a]
		Weed cover	93 [b]	15	56 [bc]	51	0 [a]
		Imidacloprid	102 [b]	19	128 [c]	32	1 [a]
		Untreated control	95 [b]	26	80 [bc]	40	53 [b]
	Inverleigh 2010	Broad-spectrum	60	-	0	11	6
		Thiamethoxam/abamectin	75	-	4	7	8
		Dimethoate	53	-	3	3	6
		Weed cover	112	-	1	26	6
		Imidacloprid	98	-	5	6	4
		Untreated control	27	-	1	11	4
	Rossbridge 2010	Broad-spectrum	54 [a]	-	75 [a]	63 [a]	57 [a]
		Diafenthiuron	75 [ab]	-	146 [a]	91 [a]	105 [ab]
		Thiamethoxam/abamectin	52 [a]	-	90 [a]	124 [a]	110 [ab]
		Weed cover	278 [b]	-	676 [b]	662 [b]	585 [c]
		Imidacloprid	46 [a]	-	123 [a]	92 [a]	72 [ab]
		Untreated control	99 [ab]	-	113 [a]	111 [a]	168 [b]

Table S2. Average number of non-target invertebrates collected from pitfall samples and results from one-way ANOVAs comparing treatments at each field site. PreBE or post sowing sampling dates were used as covariates in the analyses. Different letters indicate significantly different means at each sampling date (at the $p < 0.05$ level, Tukey's-*b post hoc* test).

Functional Group	Trial Site	Treatment	0-DAT	14-DAT	35-DAT
Formicidae	Inverleigh 2009	Broad-spectrum	4	3	2 [a]
		Thiamethoxam/abamectin	9	8	7 [b]
		Spinosad	13	6	6 [b]
		Weed cover	5	4	2 [a]
		Imidacloprid	14	5	5 [b]
		Untreated control	13	7	6 [b]
	Rossbridge 2009	Broad-spectrum	1 [a]	1	2 [a]
		Diafenthiuron	1 [a]	5	7 [ab]
		Paraffinic oil	3 [ab]	4	8 [ab]
		Weed cover	2 [a]	1	3 [ab]
		Imidacloprid	2 [ab]	6	6 [ab]
		Untreated control	4 [b]	5	10 [b]

Table S2. *Cont.*

Functional Group	Trial Site	Treatment	0-DAT	14-DAT	35-DAT
Formicidae	Inverleigh 2010	Broad-spectrum	7	1 [a]	1 [a]
		Thiamethoxam/abamectin	5	1 [ab]	1 [ab]
		Dimethoate	9	4 [c]	2 [ab]
		Weed cover	7	4 [c]	2 [ab]
		Imidacloprid	9	4 [c]	3 [b]
		Untreated control	7	2 [bc]	2 [ab]
	Rossbridge 2010	Broad-spectrum	2	2	1 [a]
		Diafenthiuron	1	3	2 [b]
		Thiamethoxam/abamectin	1	2	2 [ab]
		Weed cover	2	2	1 [a]
		Imidacloprid	1	3	2 [b]
		Untreated control	2	3	3 [b]
Predatory beetles	Inverleigh 2009	Broad-spectrum	7 [ab]	18	10 [a]
		Thiamethoxam/abamectin	7 [ab]	19	4 [ab]
		Spinosad	8 [a]	34	6 [ab]
		Weed cover	3 [b]	16	2 [b]
		Imidacloprid	14 [a]	40	4 [ab]
		Untreated control	9 [a]	36	4 [ab]
	Rossbridge 2009	Broad-spectrum	1 [a]	2	11
		Diafenthiuron	3 [bc]	4	42
		Paraffinic oil	1 [abc]	4	28
		Weed cover	1 [ab]	2	13
		Imidacloprid	5 [c]	4	27
		Untreated control	2 [abc]	3	23
	Inverleigh 2010	Broad-spectrum	2	2 [a]	1
		Thiamethoxam/abamectin	2	3 [ab]	1
		Dimethoate	3	6 [b]	1
		Weed cover	3	3 [ab]	2
		Imidacloprid	3	5 [ab]	2
		Untreated control	3	6 [b]	3
	Rossbridge 2010	Broad-spectrum	4	8	74
		Diafenthiuron	5	9	87
		Thiamethoxam/abamectin	3	7	92
		Weed cover	4	16	85
		Imidacloprid	4	11	79
		Untreated control	4	15	81

© 2015 by the authors; licensee MDPI, Basel, Switzerland. This article is an open access article distributed under the terms and conditions of the Creative Commons Attribution license (http://creativecommons.org/licenses/by/4.0/).
